# An Intrusion Detection System Based on Multi-Level Clustering for Hierarchical Wireless Sensor Networks

**DOI:** 10.3390/s151128960

**Published:** 2015-11-17

**Authors:** Ismail Butun, In-Ho Ra, Ravi Sankar

**Affiliations:** 1 Department of Mechatronics Engineering, Bursa Technical University, Gaziakdemir M. Mudanya C. No:4/10, Osmangazi 16190, Turkey; 2 Department of Electrical Engineering, University of South Florida, 4202 E. Fowler Avenue, Tampa, FL 33620, USA; E-Mail: sankar@usf.edu; 3 School of Computer, Information and Communication Engineering, Kunsan National University, Gunsan 573-701, Korea

**Keywords:** intrusion detection, neighborhood watching, clustering, WSN, IDS, hierarchical

## Abstract

In this work, an intrusion detection system (IDS) framework based on multi-level clustering for hierarchical wireless sensor networks is proposed. The framework employs two types of intrusion detection approaches: (1) “downward-IDS (D-IDS)” to detect the abnormal behavior (intrusion) of the subordinate (member) nodes; and (2) “upward-IDS (U-IDS)” to detect the abnormal behavior of the cluster heads. By using analytical calculations, the optimum parameters for the D-IDS (number of maximum hops) and U-IDS (monitoring group size) of the framework are evaluated and presented.

## 1. Introduction and Related Work

A group of sensing devices team up to form a wireless network to be named as wireless sensor network (WSN). WSNs constitute a challenging research area of engineering and still continue to attract scientists who are working in the following application areas: monitoring physical phenomena, such as fire, earthquakes, pollution; gathering information in military operations, such as reconnaissance and surveillance; gathering information for healthcare systems; building and construction safety; manufacturing machinery performance; *etc* [1,2].

WSNs have specific challenges due to low transmission bandwidth, constrained power supplies, tiny memory sizes and, finally, limited power [3,4]. This brings the fact that the security solutions devised for traditional networks cannot be applied to WSNs directly [5]. Therefore, new ideas and approaches (algorithms) are needed in order to increase the security of WSNs. In this paper, we propose security enhancements (especially on intrusion detection) for WSNs that are deployed in a hierarchical topology (clustered).

Intrusion is an active (e.g., packet dropping) or passive (e.g., eavesdropping) activity in a network without having permission to do so. While securing networks, “intrusion prevention” constitutes the first and “intrusion detection” constitutes the second line of defense. If authorized network members represent any misbehavior (e.g., packet dropping), intrusion prevention systems cannot stop this activity. Therefore intrusion detection systems come into play.

**Figure 1:**
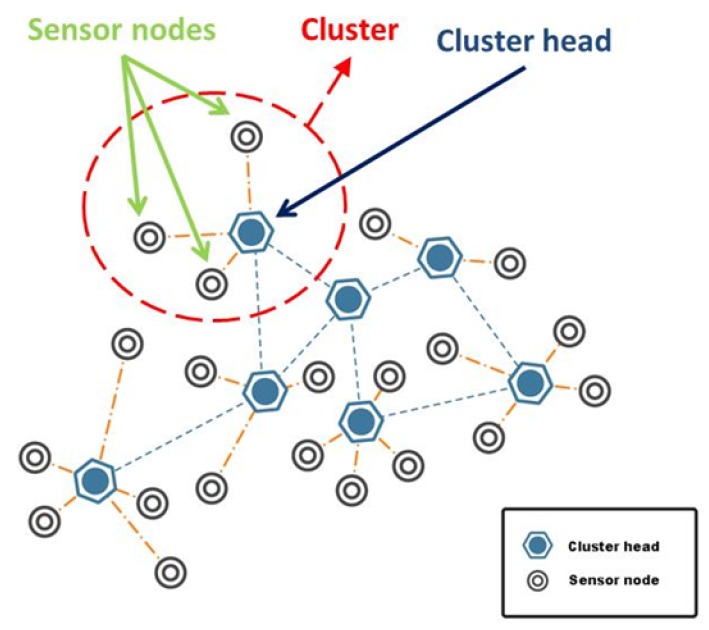
A typical clustered WSN.

A typical hierarchical WSN consists of clusters, as shown in Figure 1. Each cluster is a group of interconnected sensor nodes with a dedicated node called the cluster head (CH). CHs are responsible for managing the member (subordinate) sensor nodes; such as scheduling of the medium access, dissemination of the control messages and, most importantly, data aggregation. Clustering methods devised for WSNs are out of the scope of this paper. Interested readers may refer to Abbasi and Younis’s work [6] and Butun *et al.*’s work [7], as they provide thorough literature surveys in the mentioned field.

**Figure 2:**
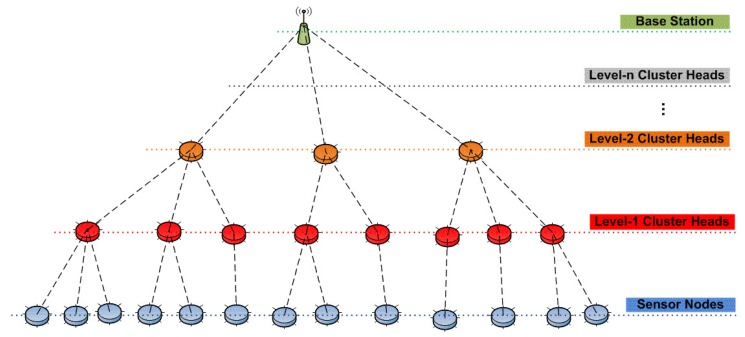
Multi-level clustering for our proposed IDS framework.

In some practical configurations of hierarchical WSNs, CHs may also form a higher level of cluster in which one of the CHs is assigned as the CH (Level 2) of all other CHs (Level 1). This leveled architecture is shown in Figure 2. In a leveled and clustered WSN; the detection of abnormal behaviors of bottom level nodes (sensor nodes) is not enough to detect all of the intrusions of the network. This is because of the fact that CHs and upper level clusters may also be compromised. Following the deployment of the nodes and the formation of the clusters and the CHs, CHs may constitute a single point of failure. Therefore, in order to have a complete intrusion detection system (IDS) for hierarchical WSNs, intrusions through CHs need to be detected, as well.

It is important to emphasize that our focus in this research is on the hierarchical WSNs, meaning that sensor nodes are gathered into groups called “clusters”. Here, we will discuss only the references that are directly related to our paper. In the IDS approaches proposed by [8,9,10], the direction of the alert propagation is from subordinates through CHs, leaving the following question unanswered for the detection part: “What happens if a malicious upper level CH (Level 2) drops the packet that is coming from a member (subordinate) node (Level 1) and is about to alert an upper level CH (Level 3) concerning the misbehavior of the mentioned CH (Level 2)?”. This is an open gap in the security of the mentioned schemes. In the IDS approaches proposed by Agah *et al.* [11,12], only one of the clusters of the network is monitored at a time. This leaves the rest of the network vulnerable. In the IDS approach of Su *et al.* [13], both downward and upward protections are provided. This means that CHs monitor subordinate nodes for downward protection and subordinate nodes monitor CHs for upward protection. However, the proposed scheme uses symmetric key cryptography, and therefore, new nodes cannot be added to the network after the deployment, which makes the proposed scheme impracticable for use.

For our IDS framework, we adopt the idea of downward and upward protection proposed by Su *et al.* [13]. For our downward (D)-IDS scheme, we adopted the “isolation table” concept that was suggested by Chen *et al.* [9] and also the “watchdog” concept that was suggested by Krontiris *et al.* [14]. For our upward (U)-IDS scheme, we adopted the “monitoring group” concept that was presented by Su *et al.* [13]. For both D-IDS and U-IDS schemes, as a detection algorithm, we adopt the “sequential probability ratio test (SPRT)” algorithm that was proposed by Brown and Du [15]. Finally, for the decision making step, the “decision engine” algorithm is used, which was proposed by Patcha *et al.* [16]. Table 1 provides an overview of our proposed IDS framework. Our proposed framework is one of a kind; hence, it provides all of the required building steps of an IDS scheme for hierarchical WSNs.

**Table 1:** Our proposed IDS framework for WSNs.

Proposed IDS Framework		
	D-IDS	U-IDS
Detection methodology	Isolation table [9]	Monitoring group [13]
	Watchdog [14]	
Detection algorithm	SPRT [15]	
Decision making	Decision engine [16]	

The rest of the paper is organized as follows: Section 2 presents the system model of the proposed IDS framework. Section 3 and Section 4 present the details of the D-IDS and U-IDS schemes, respectively. Details of the SPRT that is used in our proposed D-IDS and U-IDS schemes is presented in Section 5.1, whereas Section 5.2 summarizes the decision making process following the SPRT for each scheme. In Section 6, the effect of cluster size (maximum hops between cluster head and cluster members) on the detection probability of our intrusion detection system is investigated, when the IDS is located on the CH (D-IDS). In the reverse manner, in Section 7, the effect of the total number of monitoring nodes on the detection probability of a malicious cluster head is investigated, when the IDS is located on the member nodes of a cluster (U-IDS). Finally, Section 8 concludes the paper.

## 2. System Model

As the name implies, the proposed IDS is based on multi-level clustering, meaning that Level 1 CHs are the CHs for sensor nodes and at the same time subordinates for Level 2 CHs, in the same manner, Level 2 CHs are the CHs for Level 1 CHs and at the same time subordinates for Level 3 CHs, and so on, as shown in Figure 2.

For the selection of the cluster heads, our proposed power and connectivity-aware clustering algorithm (for details, refer to [7,17]) can be used as follows:

- Level 1 CHs would be selected by selecting the maximum hop size as “1”.
- Level 2 CHs would be selected by selecting the maximum hop size as “2”.
- …
- Level-*n* CHs would be selected by selecting the maximum hop size as “*n*”.

Our proposed IDS framework provides two types of intrusion detection approaches for the presented multi-level clustered WSN:

1. Downwards intrusion detection system (D-IDS): CHs are responsible for monitoring all of the activities of their subordinates by using watchdogs and recording their activities in a table called the “isolation table”.
2. Upwards intrusion detection system (U-IDS): A certain number of (monitoring group size, *m*) subordinates coordinately monitor the activity of the CH and report any abnormal activity to an upper level CH.

Intrusions through subordinates of the network are detected by the D-IDS, and intrusions through CHs of the network are detected by the U-IDS. In this way, our overall proposed IDS framework (D-IDS and U-IDS) covers the entire network in terms of detecting intrusions.

## 3. Downwards Intrusion Detection System (D-IDS)

**Figure 3:**
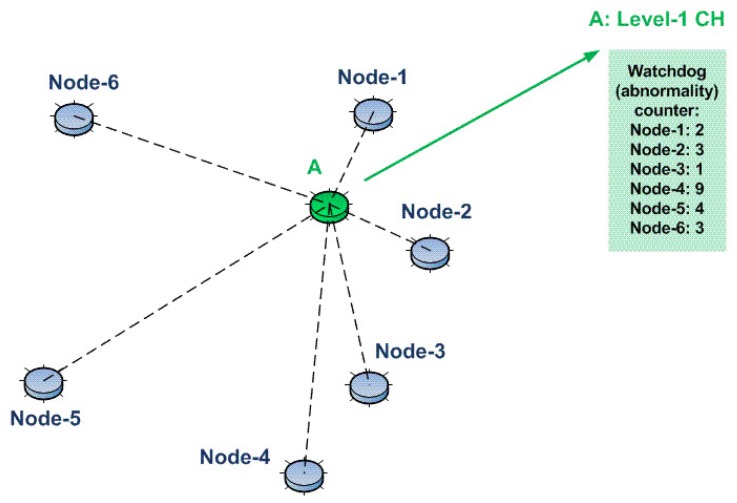
The usage of the watchdog counter concept for our D-IDS.

CHs hold watchdog counters with abnormality counters for each subordinate. Since the intrusion detection direction is from CHs towards subordinates, we call this scheme D-IDS. For example, consider the network shown in Figure 3. Here, Node A is the Level 1 CH of the remaining nodes. Therefore, it has a watchdog counter (that counts the pre-defined abnormalities) for each subordinate node, namely Node 1, Node 2, …, Node 6.

**Figure 4:**
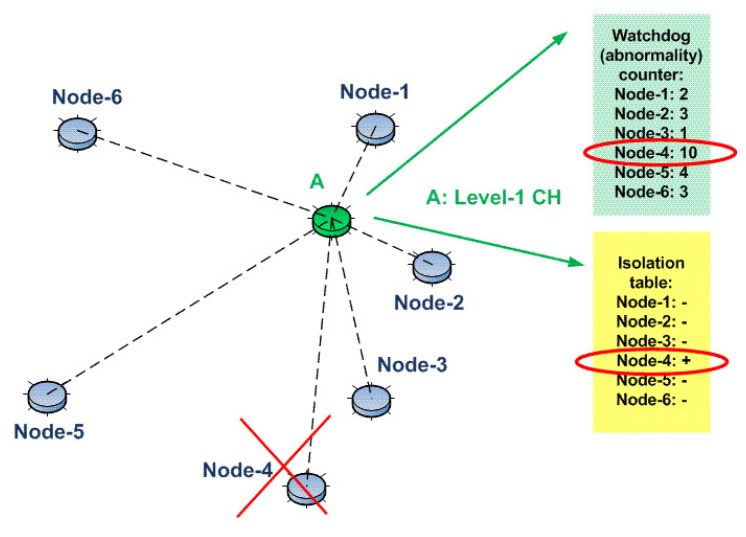
The usage of the isolation table concept in our D-IDS.

Whenever any watchdog counter reaches a certain threshold, the associated node is flagged and included in the isolation table. Then, as a mitigation step, any communication with this node is blocked (packets to be forwarded to this node, as well as the packets coming from this node are dropped). For example, consider again the same network shown in Figure 3. However, this time, assume that the watchdog counter of Node 4 is “10” and also assume that the threshold level for the watchdog counters is “10”, as well. Since the watchdog counter of Node 4 has reached the threshold, Node 4 is marked as an abnormal node in the isolation table, as shown in Figure 4. Then, finally, all communications with Node 4 are blocked by the Level 1 CH (Node A).

**Figure 5:**
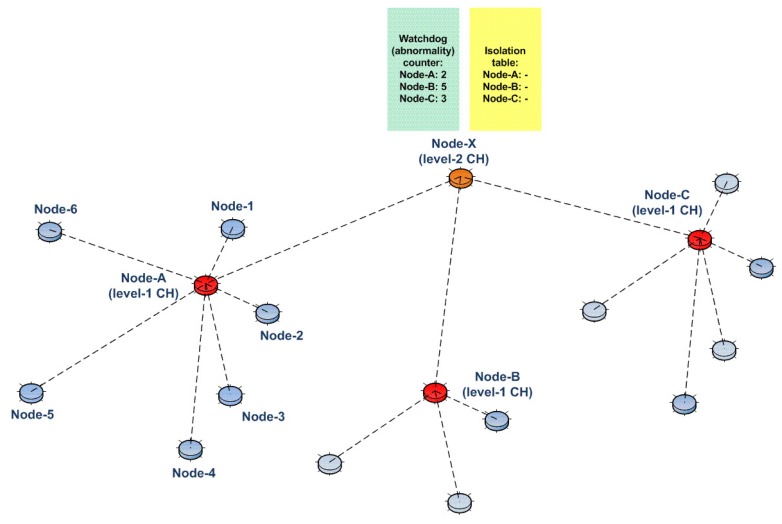
Implementation of D-IDS for upper levels of the network.

The mentioned D-IDS is applicable to all levels. For example, consider the network shown in Figure 5. This time, Node A is a subordinate of Node X (an upper level node, Level 2), just like Node B and Node C. Node X holds watchdog counters for the abnormal behaviors of Node A, Node B and Node C. As mentioned above, if any watchdog counter reaches a certain threshold, the associated entry in the isolation table will be marked, and a mitigation technique is issued (revocation of the node from the network).

## 4. Upwards Intrusion Detection System (U-IDS)

A certain number of (monitoring group size, *m*) subordinates coordinately monitor the activity of the CH and report any abnormal activity to an upper level CH. Abnormal activity is determined when the watchdog counter reaches or exceeds a certain threshold. In accordance with the coordination concept, the total value of the abnormal cases is calculated by the logical “OR(+)” operation; individual watchdog results of each monitoring node are OR’ed with the rest of the watchdog results. As a result, the final decision associated with the abnormal behavior of the CH is concluded by the coordinated effort of all monitoring nodes.

The rationale for using the OR operation is as follows: In some specific time interval, some of the monitoring nodes might be in “sleep mode”, and therefore, these nodes might miss the abnormal behavior of the CH. However, in that specific time frame, the other monitoring nodes possibly would be in “awake mode” and catch the incidence. Therefore, after a certain period of time (update interval), each monitoring node sends its individual result to the rest of the monitoring nodes, and a final decision is made.

In order to catch most of the incidences (high probability of detection), the sleep/awake cycles of the monitoring nodes should be assigned accordingly. For instance, if there are three monitoring nodes in a cluster and if one of them is in sleep mode in a specific time frame, then in order to catch the incidences, the rest of the monitoring nodes should be in awake mode.

**Figure 6:**
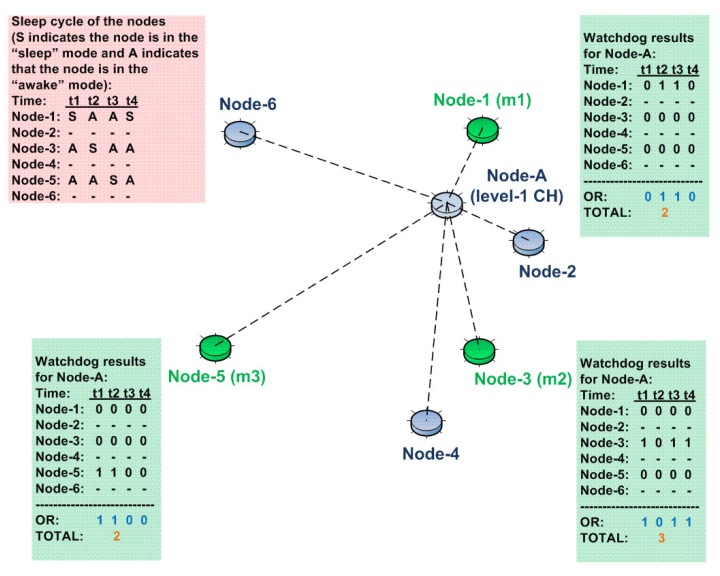
The usage of the monitoring group concept in our U-IDS.

Consider the cluster of a network shown in Figure 6. Here, Node A is again the Level 1 CH of the remaining nodes. Subordinate nodes are, namely, Node 1, Node 2, …, Node 5 and Node 6. Among those, Node 1, Node 3 and Node 5 constitute the monitoring group. Their responsibility is to monitor the abnormal activity of Node A (consider that Node A is showing abnormal behavior for all time frames $t_{1},t_{2},t_{3},t_{4}$). At a specific time frame, if the monitoring nodes are in awake mode and detect any abnormality, they update their watchdog counters, accordingly. For example, at the specific time frame of $t_{1}$, Node 1 was in sleep mode, and therefore, it was not able to detect any abnormality. However, Node 3 and Node 5 were in awake mode, detected an abnormality of Node A and updated their own watchdog counters, accordingly (associated with the time frame of $t_{1}$). At the end of the time frame $t_{4}$, it is observed that out of four instances, Node 1 detected two instances, Node 3 three instances and Node 5 two instances.

**Figure 7:**
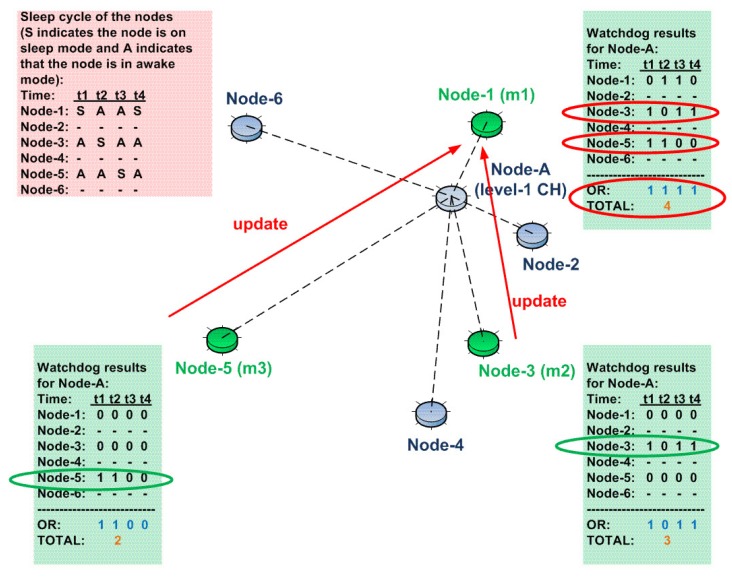
Watchdog update propagation in our U-IDS.

The monitoring nodes share the entries of their watchdog counters among each other in certain time intervals. In the specific case shown in Figure 6, assume that the monitoring nodes share the entries of their watchdog counters after the time frame $t_{4}$. This is shown in Figure 7. Here, as a result of the arrival of the watchdog counter entries from Node 3 and Node 5, Node 1 (*m1*) updates the watchdog counter entries associated with them, and finally, the total number of encounters is calculated by the OR operation, as mentioned earlier. In accordance with the updates from Node 3 and Node 5, Node 1 has refreshed its watchdog counter and calculated the total number of incidences as “4”. In the same manner, Node 3 and Node 5 will update their watchdog counter entries and calculate the total number of incidences as “4”.

After each update interval, monitoring nodes check their updated counter values. Whenever their watchdog counter reaches a certain threshold (“15” in our example), they send an encrypted alert message to an upper level CH. The reason for the encryption is to hide the alert message from the CH that is under investigation (Node A in our example).

These alert messages are sent directly to the upper level cluster head. In order to do so, we assume that the radios of the nodes have two different operation modes: “normal mode” and “alert mode”. In the normal mode, since CHs are generally one hop away, the radios operate to transmit in a short range. This energy-saving mode helps nodes to increase their life time. In the alert mode, the radios operate to transmit in a long range. In this way, without the help of intermediate nodes, a monitoring node can directly send the alert messages to an upper level CH.

**Figure 8:**
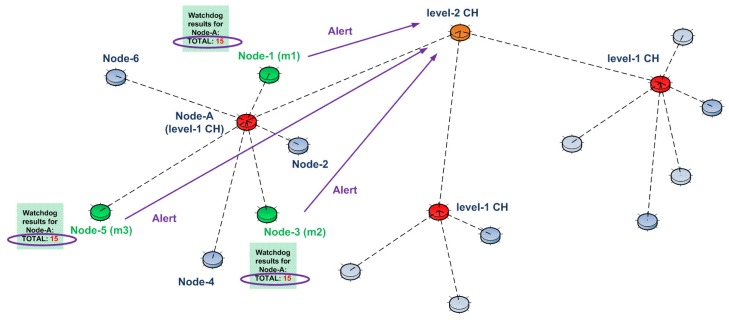
Alert propagation towards upper levels in our U-IDS.

Consider the case shown in Figure 8. Here, as mentioned above, in each update interval, each monitoring node updates the other monitoring nodes and also check its watchdog counter. After a certain period of time, watchdog counters of all of the monitoring nodes reach the threshold value of “15”. Therefore, they change their radio’s mode of operation to “alert mode” and send an encrypted alert message directly to an upper level CH, namely a Level 2 CH. In this specific example, Node 1 has a higher probability to alert Level 2 CH, since its location is closer than Node 3 and Node 5.

## 5. Detection of DoS Attacks in WSNs by Using SPRT

Wald’s sequential probability ratio test (SPRT) for the detection of selective forwarding attacks (a denial of service (DoS) attack in the network layer of WSNs) was used in [15]. This method detects intentional packet drops with a high probability of detection rate. Therefore, it can be applied to any packet drop attack towards the security of WSNs, for example DoS attacks in the network layer (black hole attacks, sinkhole attacks, *etc.*).

### 5.1. SPRT

**Figure 9:**
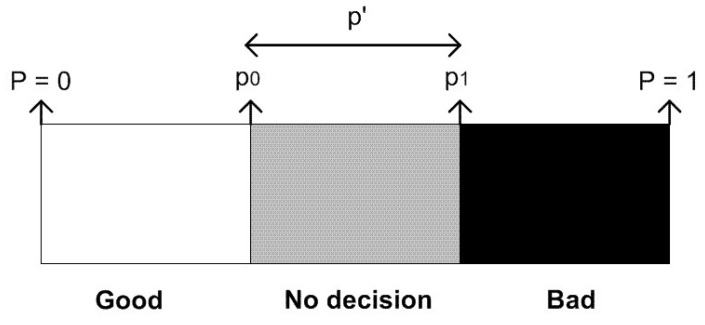
Thresholds for the decision making.

According to SPRT, a random variable *p* is used to define the status of packet forwarding, where 0 denotes successful transmission of the packet (good) and 1 denotes a packet drop (bad). *p* is calculated as the percentage of dropped packets over all packets to be transmitted. $p^{\prime }$ is defined as the acceptable probability of dropped packets. A node is considered as legitimate if $p\leq p^{\prime }$ holds, and it is considered as compromised if $p>p^{\prime }$ holds. $p_{0}<p^{\prime }<p_{1}$ defines the “gray region” for the decision making, where the decision is inconclusive regarding the legitimacy of the node. Figure 9 illustrates the decision boundaries for the SPRT, namely; white, gray and black regions.

Note that 1,2,…,m represents the sample number. The ultimate goal is to minimize the missed detection rate, $\alpha =P_{1}\left(|D_{m}=0\right)$ and the false alarm rate, $\beta =P_{0}\left(|D_{m}=1\right)$, where $D_{m}$ stands for the decision at step *m* ($P_{1}$ denotes that a failure occurred and the packet is dropped; $P_{0}$ denotes that the packet is transmitted successfully; whereas, $\left(D_{m}=0\right)$ means that the decision of the SPRT for the packet transmission is positive (the packet is concluded to be transmitted); and $\left(D_{m}=0\right)$ means that the decision is negative (the packet is concluded to be dropped)). At the same time, we need to achieve this in the minimum number of samples ($m_{min}$). SPRT calculates this number as shown in Equation (1):

(1) $$m_{min}=\frac{L(p)\log(\beta )+(1-L(p))\log(\alpha )}{p\log\left(\frac{p_{1}}{p_{0}}\right)+\left(1-p\right)\log\left(\frac{1-p_{1}}{1-p_{0}}\right)}$$

where L(p) is calculated as shown in Equation (2),

(2) $$L\left(p\right)=\frac{{\left(\frac{1-\beta }{\alpha }\right)}^{h}-1}{{\left(\frac{1-\beta }{\alpha }\right)}^{h}-{\left(\frac{\beta }{1-\alpha }\right)}^{-h}}$$

and *h* can be determined by solving Equation (3):

(3) $$p=\frac{1-{\left(\frac{1-p_{1}}{1-p_{0}}\right)}^{h}}{{\left(\frac{p_{1}}{p_{0}}\right)}^{h}-{\left(\frac{1-p_{1}}{1-p_{0}}\right)}^{h}}$$

After setting all of the parameters ($p_{0},p_{1},\alpha ,\beta$) and collecting *m* samples, the acceptance threshold ($a_{m}$) and the rejection threshold ($r_{m}$) can be found by using Equations (4) and (5), respectively:

(4) $$a_{m}=\frac{\log(\frac{\beta }{1-\alpha })}{\log\left(\frac{p_{1}}{p_{0}}\right)-\log\left(\frac{1-p_{1}}{1-p_{0}}\right)}+m\frac{\log(\frac{1-p_{0}}{1-p_{1}})}{\log\left(\frac{p_{1}}{p_{0}}\right)-\log\left(\frac{1-p_{1}}{1-p_{0}}\right)}$$

(5) $$r_{m}=\frac{\log(\frac{1-\beta }{\alpha })}{\log\left(\frac{p_{1}}{p_{0}}\right)-\log\left(\frac{1-p_{1}}{1-p_{0}}\right)}+m\frac{\log(\frac{1-p_{0}}{1-p_{1}})}{\log\left(\frac{p_{1}}{p_{0}}\right)-\log\left(\frac{1-p_{1}}{1-p_{0}}\right)}$$

Let $\left\lceil a_{m}\right\rceil$ denote the upper bound for $a_{m}$ and $\left\lfloor r_{m}\right\rfloor$ denote the lower bound for $r_{m}$. For each sample, $\left\lceil a_{m}\right\rceil$ and $\left\lfloor r_{m}\right\rfloor$ should be revised according to new values of $a_{m}$ and $r_{m}$, respectively.

Let $d_{m}$ denote the number of packets dropped in the *m* number of samples. Then, the SPRT test needs to be continuously performed as long as Equation (6) holds:

(6) $$\left\lceil a_{m}\right\rceil <d_{m}<\left\lfloor r_{m}\right\rfloor$$

At each round of the SPRT test, there are three possible outcomes based on the result of the comparison shown in Equation (6):

1. If $d_{m}\leq \left\lceil a_{m}\right\rceil$, then the conclusion is that the node is legitimate.
2. If $\left\lceil a_{m}\right\rceil <d_{m}<\left\lfloor r_{m}\right\rfloor$, then the SPRT test needs to be continued.
3. $\left\lfloor r_{m}\right\rfloor \leq d_{m}$, then the conclusion is that the node is compromised.

### 5.2. Decision Making Process of IDS Following the SPRT

According to Patcha *et al.* [16], the decision engine (*i.e.*, decision making algorithm) of an IDS concludes either one of four decisions (with non-zero probabilities) as a result of the decision making process over a triggered alarm (event):

- Intrusive, but not anomalous (false-negative): there is an intrusion into the system, but IDS fails to detect it and concludes the event to be a non-anomalous one.
- Not intrusive, but anomalous (false-positive): there is no intrusion into the system, but IDS mistakenly concludes a normal event to be an anomalous one.
- Not intrusive and not anomalous (true-negative): there is no intrusion into the system, and IDS concludes the event to be an non-anomalous one.
- Intrusive and anomalous (true-positive): there is an intrusion into the system, and IDS concludes the event to be an anomalous one.

**Figure 10:**
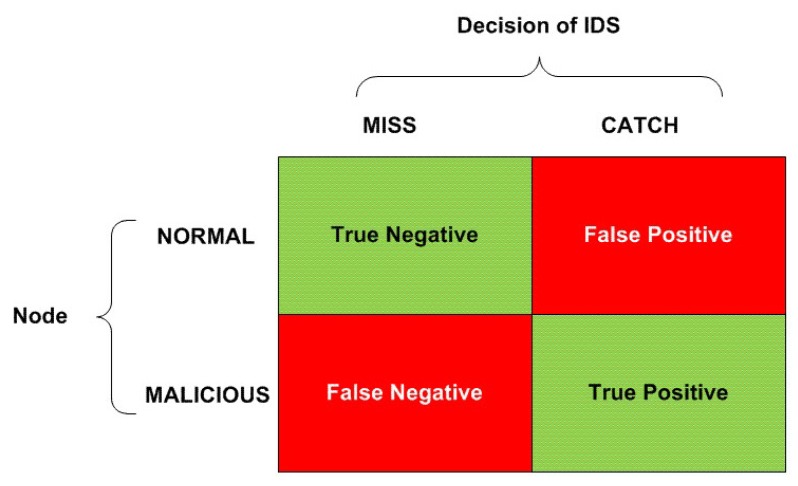
All possible detection results of an IDS.

Figure 10 summarizes the mentioned possibilities regarding the legitimacy assessment of a node. Eventually, we expect the selected decision making algorithm to generate a greater percentage of true-positives and true-negatives and a lesser percentage of false-positives and false-negatives.

## 6. The Effect of Cluster Size on the Detection Probability of the D-IDS

In this section, we followed Shin *et al.*’s approach [8] to evaluate the effect of clustering on the detection probability of D-IDS. Our main focus is to calculate the effect of the maximum distance (hops) between CH and cluster members on the intrusion detection probability.

**Figure 11:**
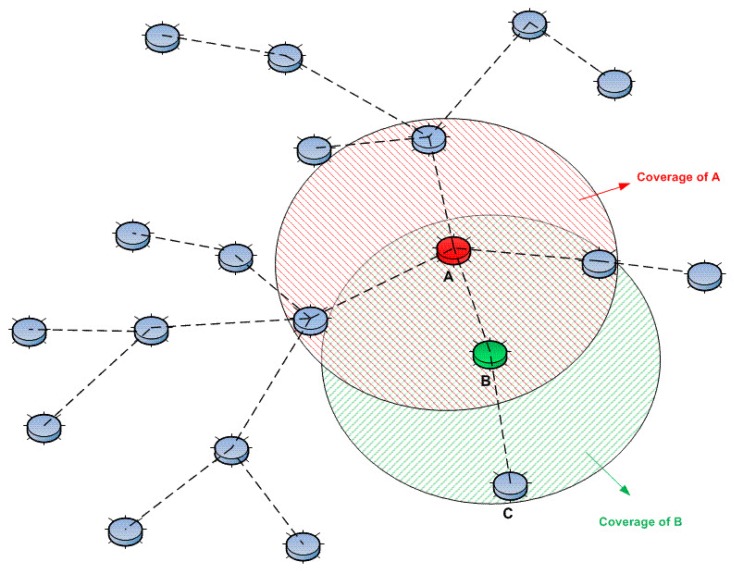
An example of a clustered network with a maximum hop distance of two.

In Figure 11, a WSN is divided into clusters. The maximum distance of each cluster is two hops, meaning that in a cluster, a CH (denoted as red nodes in the figure) is a maximum of two hops away from its member nodes. As an example, node *A* is a CH, and nodes *B* and *C* are its member nodes. A two-hop cluster does not mean that node *C* is in direct communication range of *A* (as we can see in the figure, *C* is outside of the communication range of *A*), but it means that *A* can connect and monitor the behavior of *C* through node *B* (since *B* is in communication range of both *A* and *C*, *B* performs as a relaying node between nodes *A* and *C*).

CHs (such as node *A*) can use indirect monitoring (*A* monitors node *C* through node *B*) through intermediate nodes (such as node *B*) to detect intrusions that would happen at the end nodes (such as node *C*). However, this kind of monitoring definitely will increase the network overhead. Besides, the reliability of the intermediate nodes, such as the sleep rate and error rate, would certainly change the overall performance of the intrusion detection system.

Let us define the average sleep rate and the average error rate of all of the nodes (except the CH and the malicious node) to be *s* and *e*, respectively. Here, the error rate is due to packet losses caused by transmission problems, such as packet collisions. We assume the probability distributions of these random variables (*s* and *e*) to be Gaussian with means 0≤E(s)≤1 and 0≤E(e)≤1 and variances $\sigma_{s}^{2}$ and $\sigma_{e}^{2}$.

If a CH detects a malicious node that is located at *j*-hops away from the CH in an *i*-hops cluster, where *i* is the cluster size in terms of the number of hops, and if 0≤j≤i holds, then the detection probability ($p_{i,j}$) of each node is given by:

(7) $$p_{i,j}={\left[\left(1-E\left(s\right)\right)\left(1-E\left(e\right)\right)\right]}^{j-1}$$

In Table 2, different values of $p_{i,j}$ are shown as *i* and *j* are varied. Here, note that the value of *j* cannot exceed the value of *i*.

**Table 2:** Detection probability ($p_{i,j}$) for different values of *i* and *j*.

	j=1	j=2	j=3	j=4
i=1	1	N/A	N/A	N/A
i=2	1	(1−E(s))(1−E(e))	N/A	N/A
i=3	1	(1−E(s))(1−E(e))	${\left[\left(1-E\left(s\right)\right)\left(1-E\left(e\right)\right)\right]}^{2}$	N/A
i=4	1	(1−E(s))(1−E(e))	${\left[\left(1-E\left(s\right)\right)\left(1-E\left(e\right)\right)\right]}^{2}$	${\left[\left(1-E\left(s\right)\right)\left(1-E\left(e\right)\right)\right]}^{3}$

Finally, the average detection probability ($P_{i}$) of the *i*-hops cluster is given by:

(8) $$P_{i}=\frac{1}{i}\sum_{j=1}^{i}p_{i,j}=\frac{1}{i}\sum_{j=1}^{i}{\left\{\left(1-E\left(s\right)\right)\left(1-E\left(e\right)\right)\right\}}^{j-1}$$

**Figure 12:**
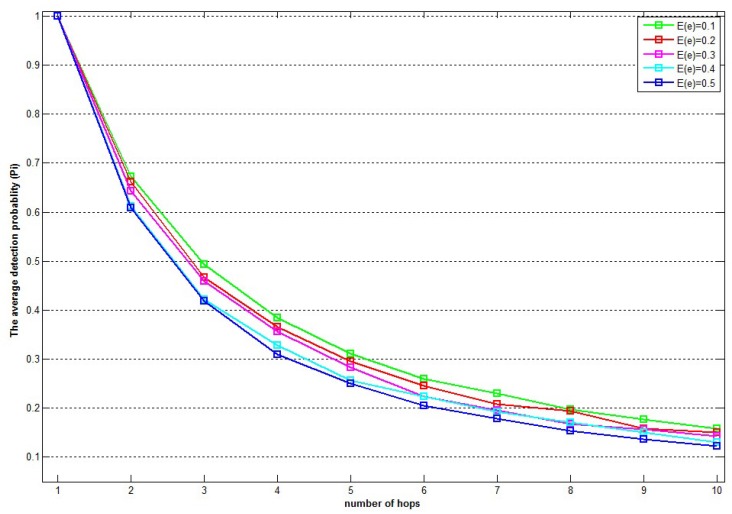
The effect of cluster size on the detection probability of the D-IDS for various packet loss rates while the sleep rate is 60%.

**Figure 13:**
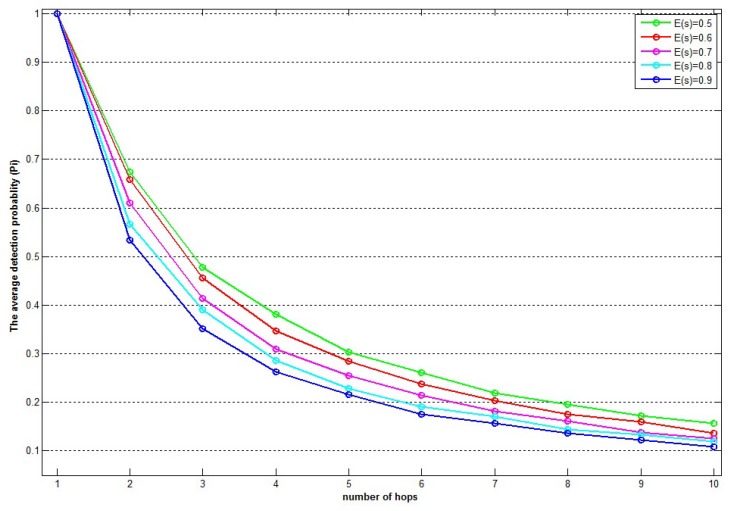
The effect of cluster size on the detection probability of the D-IDS for various sleep rates while the packet loss rate is 30%.

Figure 12 and Figure 13 show different values of $P_{i}$ as the maximum number of hops (*i*) changes according to Equation (8). There are five plots in each figure, for various values of E(e) in Figure 12 and E(s) in Figure 13, respectively.

In Figure 12, E(s) is fixed at E(s)=0.6, meaning that the average sleep rate of the nodes is 60%. Each plot represents a different value of E(e) with a variance of 10%. From the plots, it can be observed that as the packet loss rate of each node increases, the average intrusion detection probability of the D-IDS decreases, which is expected. As the error rate increases, it becomes difficult for the D-IDS to determine if the loss of a packet was caused by a channel error (natural causes) or an outside (intruder) effect.

In Figure 13, E(e) is fixed at E(e)=0.3, meaning that the average packet drop rate of the nodes is 30%. Each plot represents a different value of E(s) with a variance of 10%. From the plots, it can be observed that as the sleep rate of each node increases, the average intrusion detection probability of the D-IDS decreases, which is also expected. As the sleep rate increases, the chance of the D-IDS for catching an intrusion decreases. Hence, the fewer the number of nodes that are awake in the network, the less intrusions will be caught by the D-IDS. In other words, an intrusion that would be caught by an intermediate node would be simply missed, because the node was sleeping at the exact time that the intrusion happened.

From the plots of Figure 12 and Figure 13, we can conclude that as the number of the maximum range of a cluster (maximum number of hops from a CH and a member node in a cluster) increases, the average intrusion detection probability of the D-IDS decreases. In other words, indirect monitoring of the intrusions has a bad effect on the overall intrusion detection probability of the IDS.

## 7. The Effect of Monitoring Group Size on the Detection Probability of the U-IDS

In this section, we follow Yang *et al.*’s work [18] to investigate the effect monitoring group size (*m*) on the detection probability of malicious cluster heads in our U-IDS. Here, we assume that *m* of the member nodes of a cluster have an intrusion detection scheme that is running in a collaborative manner. Thus, cluster members periodically give a decision regarding the trustworthiness of a cluster head.

**Figure 14:**
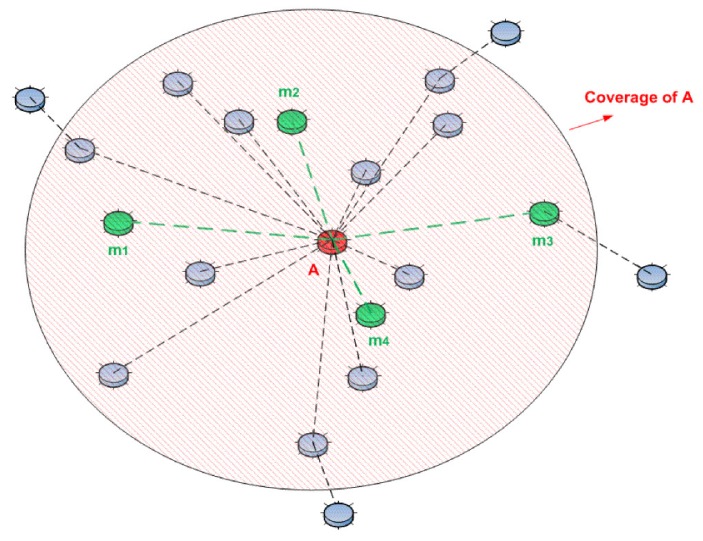
A typical 15-node clustered WSN (one-hop distance).

Consider the clustered WSN (one-hop distance) shown in Figure 14. Node *A* (marked in red color) is the cluster head, and it has 15 member nodes, which are in the radio coverage of *A*. Among these member nodes, four of them (*m = 4*; marked in green color and denoted as $m_{1}$, $m_{2}$, $m_{3}$ and $m_{4}$) are collaborating to monitor the activity of *A*.

In order to calculate the effect of monitoring group size (*m*) on the detection probability of a malicious cluster head, assume that the size of a cluster is *N* (15 in our case) and the probability of each member node to detect the malicious cluster head is $P_{d}$. Then, the total probability of the malicious cluster head to be detected $P_{D}$, as a result of the collaboration size (= monitoring group size = *m* number of the nodes) is calculated as shown in Equation (9):

(9) PD=∑k=mNNkPdk(1−Pd)N−k

**Figure 15:**
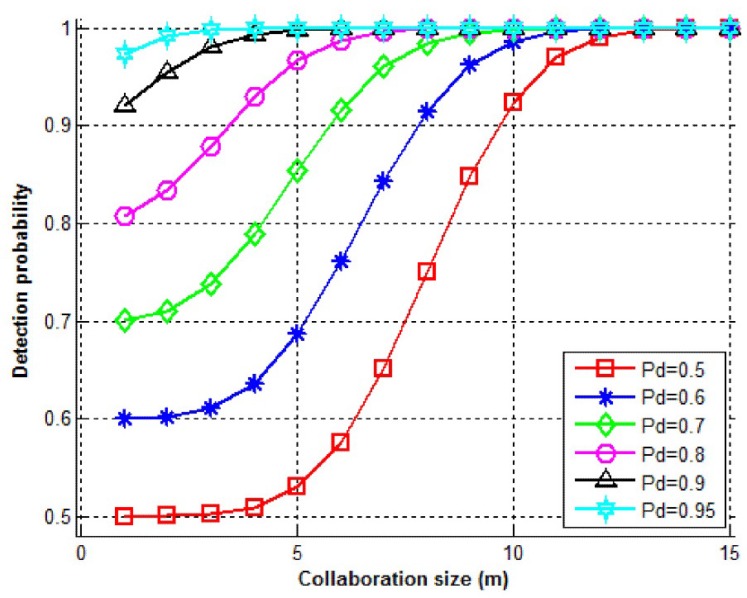
Detection probability ($P_{D}$) *vs*. collaboration size (*m*) for various values of $P_{d}$.

Figure 15 shows the change of the overall detection probability ($P_{D}$) with respect to the collaboration size for various values of $P_{d}$, for a cluster size of 15 (N=15). Accordingly, it can be observed that collaboration is useful in increasing the overall detection rate ($P_{D}$). As the collaboration size increases, overall detection probability increases and approaches one (100%).

Again, assume that the size of a cluster is *N* and that the probability of each member node to fail (false-alarm) in detecting the malicious cluster head is $P_{f}$. Then, the total probability of the malicious cluster head goes undetected (false-alarm) $P_{F}$, as a result of the collaboration size (*m* number of the nodes) being calculated as shown in Equation (10):

(10) PF=∑k=mNNk(1−Pf)k(Pf)N−k

**Figure 16:**
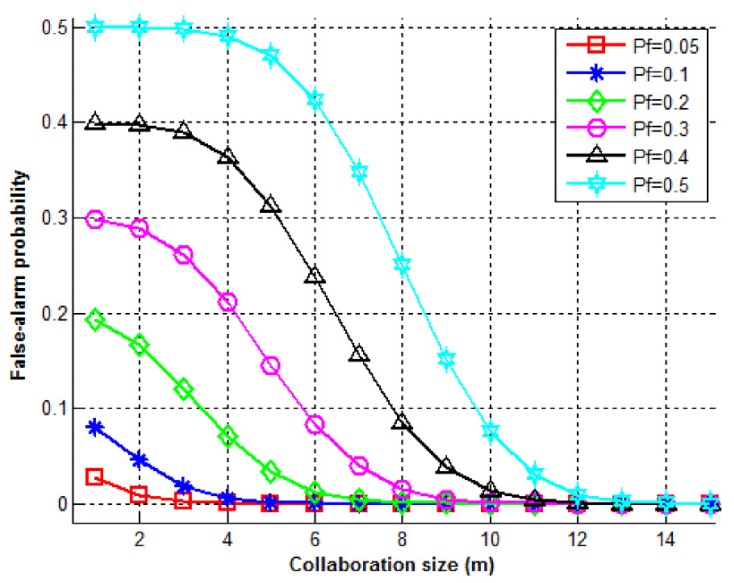
False-alarm probability ($P_{F}$) *vs*. collaboration size (*m*) for various values of $P_{f}$.

Figure 16 shows the change of overall false-alarm probability ($P_{F}$) with respect to the collaboration size for various values of $P_{f}$, for a cluster size of 15 (N=15). Accordingly, it can be observed that collaboration is useful in decreasing the overall false-alarm rate ($P_{F}$). As the collaboration size increases, the overall false-alarm probability decreases and approaches zero (0%).

## 8. Conclusions and Suggestions for Future Research

This work presents an intrusion detection system (IDS) framework based on multi-level clustering for hierarchical wireless sensor networks. Our proposed IDS framework provides two types of intrusion detection approaches, namely “downwards-IDS” to detect the abnormal behavior (intrusion) of the subordinate (member) nodes and “upwards-IDS” to detect the abnormal behavior of the cluster heads.

The effect of cluster size (maximum hops between the cluster head and cluster members) on the detection probability of a malicious node was evaluated, when the IDS is located on the CH (downwards-IDS). In the same manner, the effect of the total number of monitoring nodes on the detection probability of a malicious cluster head was evaluated, when the IDS is located on the member nodes of a cluster (upwards-IDS).

There is a trade-off between “maximum hop count” and “intrusion detection probability”. As the maximum hop count increases, the intrusion detection probability (of an IDS) decreases and *vice versa*. According to the results of the analytical calculations presented in Section 6, the following recommendations are provided for the maximum hop count: Figure 12 and Figure 13 suggest keeping the maximum hop distance lower than “4”. The maximum hop distance should be selected as “2” or “3”, depending on the “sleep rate” of the nodes and the “average packet loss rate” of the network (1.0 represents 100% probability of a node to be sleeping or a packet to be lost): if the “sleep rate” and/or “average packet loss rate” are higher than 0.7, then the maximum hop distance should be selected as “2”; otherwise, it should be selected as “3”.

As in most technologies, nothing comes for free. By using a greater number of monitoring members, higher detection rates and lower false alarm rates can be achieved. The cost for this achievement is the loss of scarce resources (e.g., energy). The security solutions should bring minimum extra packets to the transmission, since every extra packet causes the depletion of the batteries faster and causes the network to have less life time. Therefore, a proper trade-off point needs to be determined in finding the right number for the monitoring group size (*m*).

As Figure 15 in Section 7 suggests, out of 15 nodes in each cluster, by selecting *m = 7*, a very satisfactory detection probability (>95%) can be achieved if the individual detection probabilities are higher than 70%. Again, out of 15 nodes in each cluster and for the same monitoring group size (*m = 7*), Figure 16 suggests that the false-alarm probability will be lower than 5% if the individual false-alarm rates are lower than 30%.

By evaluating the performance of our proposed IDS algorithms analytically, we were able to demonstrate their benefits in detecting the intrusions towards WSNs. Evaluation of the proposed algorithms via simulation tools (ns-3 [19], *etc.*) is left as future work.

## Appendix

Table A1 summarizes the abbreviations used in the paper.

**Table A1:** List of abbreviations.

**CH**	cluster head
**D-IDS**	downwards IDS
**DoS**	denial of service
**IDS**	intrusion detection system
**SPRT**	sequential probability ratio test
**U-IDS**	upwards IDS
**WSN**	wireless sensor network
